# A Fine-Grained Pig Behavior Recognition Method Based on an Improved YOLOv8n Network

**DOI:** 10.3390/ani16213449

**Published:** 2026-11-01

**Authors:** Jialin Gao, Junhao Wang, Ping Jiang, Shaohui Tan

**Affiliations:** School of Mechanical and Electrical Engineering, Hunan Agricultural University, Changsha 410128, China; gjlgjlgjl2026@163.com (J.G.); junhaowang030804@stu.hunau.edu.cn (J.W.); 1233032@hunau.edu.cn (P.J.)

**Keywords:** behavior recognition, deep learning, fine-grained behavior, pig, precision livestock farming

## Abstract

Accurate recognition of pig behavior is important for animal health monitoring and precision livestock farming. However, fine-grained behavior recognition in group-housed pigs is challenging because of individual occlusion, large posture variation, and subtle visual differences among similar behaviors. Here, an improved YOLOv8n-based model, EPP-YOLOv8n, was developed for fine-grained pig behavior recognition. An efficient multi-scale attention module was inserted after the spatial pyramid pooling-fast module (SPPF) to enhance behavior-related feature extraction. A behavior-factorized asymmetric head (BFA-Head) was designed with scale-adaptive mixed convolution to reduce detection-head complexity, while posture- and interaction-assisted supervision was incorporated to guide fine-grained feature learning. A partial-channel mixed convolution module (P5-selective PLC-Mix) was deployed within the Backbone P5 C2f module to reduce redundant computation, followed by task-aware structured pruning with recovery training for further model compression. On a dataset comprising 2713 images and 30,695 annotated instances after training-set augmentation, EPP-YOLOv8n achieved 98.13% precision, 94.50% recall, and 89.07% mean average precision at intersection over union (IoU) thresholds from 0.50 to 0.95 (mAP@0.5:0.95), representing increases of 2.05, 1.52, and 1.64 percentage points, respectively, over YOLOv8n. Parameter count and billion floating-point operations (GFLOPs) were reduced by 14.59% and 9.70%, respectively. These results indicate that the proposed model can improve fine-grained behavior recognition while reducing model complexity, supporting automated behavior monitoring in group-housed pigs.

## Simple Summary

Continuous monitoring of pig behavior can help farmers identify changes in health and welfare and improve daily management. Manual observation is labor-intensive and may miss short or subtle behaviors, especially when pigs are housed together and partially hide one another. This study developed a lightweight computer-vision method to recognize fine-grained pig behaviors from images. The method combines information about body posture and interactions with feeders and drinkers while removing unnecessary computations to make the model more efficient. Tests on 2713 images showed that the method could accurately distinguish 10 fine-grained behaviors and identify feeders and drinkers. Compared with the baseline YOLOv8n, it required fewer model parameters and fewer calculations while maintaining high recognition performance. The results support automated, non-contact behavior monitoring in group-housed pigs and provide a practical basis for intelligent farm management.

## 1. Introduction

As large-scale and intensive pig farming expands, manual inspection and recording of pig behavior are time-consuming and labor-intensive and are influenced by observation frequency, operator experience, and subjective judgment. Pig posture, feeding, drinking, and activity are closely associated with health status and welfare. Consequently, continuous, non-contact behavior monitoring using machine vision has become an important research direction in precision livestock farming [1,2,3].

Early pig behavior recognition mainly relied on background subtraction, contour extraction, and hand-crafted visual features. With the development of deep learning, convolutional neural networks have become important tools for pig detection and behavior recognition. Chen et al. [4] systematically reviewed deep-learning-based methods for pig posture detection. Riekert et al. [5] used two-dimensional images to detect pig position and posture and investigated the applicability of deep learning to long-term continuous monitoring. Alameer et al. [6] developed an automated method for recognizing pig postures and drinking behavior and explored the potential of these behavioral measures for health monitoring. Zhuang et al. [7] developed a machine-vision system for monitoring feeding and drinking behavior in growing pigs. Ji et al. [8] proposed a method for position detection and posture recognition of group-housed pigs, enabling automatic recognition of standing, sitting, and lying postures. Luo et al. [9] further combined pig-head position with the spatial relationship between pigs and the feeding functional area to automatically recognize and quantify feeding behavior. These studies have advanced the automation of pig behavior monitoring, but most have focused on posture or specific functional behaviors; fine-grained composite behaviors formed jointly by posture and interaction state remain less explored.

To improve recognition performance in complex pig housing environments, recent studies have focused on network architecture optimization and model lightweighting. Pu et al. [10] proposed an improved YOLOv8n-based method for multi-scenario pig behavior recognition, enhancing behavior detection through feature extraction and detection-structure optimization. Zhang et al. [11] investigated lightweight pig behavior detection based on YOLOv8 to explore the trade-off between detection performance and model complexity. Luo et al. [12] combined model pruning and knowledge distillation to develop the lightweight YOLOv8-Piglet model for multi-behavior recognition in piglets, reducing computational cost while improving detection performance and demonstrating its potential for edge-device deployment. Zeng et al. [13] proposed MACA-Net, which uses Mamba-driven feature modeling and adaptive cross-layer attention to enhance feature representation for multi-behavior detection in group-housed pigs. These studies indicate that feature enhancement and model compression provide effective approaches for automated behavior recognition in complex pig housing environments. However, how to preserve local discriminative information among fine-grained behaviors while controlling model complexity remains an open issue.

Compared with general pig posture detection, fine-grained composite behavior recognition exhibits greater inter-class similarity and intra-class variation. For example, standing_drinking, standing_eating, and standing_idle share a similar overall standing posture, with behavioral differences mainly reflected in head position and local interactions with drinking devices and feeders. In contrast, sitting_drinking, standing_drinking, and lying_drinking share the same drinking attribute but differ in overall body posture. Wei et al. [14] systematically reviewed deep-learning-based fine-grained visual analysis and identified small inter-class differences and large intra-class variations as major challenges in fine-grained recognition. For group-housed pig feeding behavior recognition, Hu et al. [15] combined pose estimation with keypoint-feature discrimination to distinguish feeding behavior from non-nutritive visiting behavior, demonstrating the importance of local posture and spatial relationships between pigs and feeding areas. Lagua et al. [16] further compared the detection performance of pig posture behaviors, nutritive behaviors, and their combined tasks and found that combined behavior recognition was more challenging than recognizing posture or nutritive behavior alone, requiring simultaneous attention to overall body posture and local head–resource interactions. Therefore, effectively incorporating posture and interaction cues within a unified detection framework and reducing confusion among similar composite behaviors remain important research problems in fine-grained pig behavior recognition.

In addition, practical deployment in group-housed pig pens requires not only high behavior-recognition accuracy but also acceptable model complexity and inference efficiency. Wang et al. [17] introduced collaborative attention, weighted feature fusion, and wavelet convolution into YOLO11n and combined these components with transfer learning to improve the accuracy and efficiency of pig behavior detection in complex environments. Chen et al. [18] proposed DWEC-YOLO, which improves feature extraction and fusion for detecting common and aggressive behaviors in group-housed pigs. These studies demonstrate that network architecture optimization can improve behavior recognition in complex pig housing environments, but practical applicability is also closely related to dataset representativeness and evaluation protocols. Bhujel et al. [19] systematically surveyed public computer-vision datasets in precision livestock farming and reported limitations such as insufficient metadata and limited scene coverage. Li et al. [20] developed the PigLife dataset covering different production stages and benchmarked multiple object detection and instance segmentation algorithms, providing a reference for the development and comparison of visual models for pig farms. Therefore, for fine-grained composite behavior recognition in complex group-housed environments, it remains valuable to reduce redundant computation while preserving discriminative information from both overall posture and local interactions. In a related object detection context, Gu and Chen [21] demonstrated that training-stage auxiliary guidance could improve detection robustness without introducing additional inference overhead, as the auxiliary components were removed after training.

To address these issues, this study developed EPP-YOLOv8n, a lightweight model for fine-grained behavior recognition in group-housed pigs, based on YOLOv8n. An efficient multi-scale attention module (EMA) was inserted after the SPPF module in the backbone to enhance behavior-related feature representation. A Behavior-Factorized Asymmetric Head (BFA-Head) was designed to reduce classification-head complexity through scale-adaptive mixed convolution while using posture- and interaction-assisted supervision to guide the learning of discriminative features of composite behaviors. In addition, P5-selective PLC-Mix was deployed on the P5 path, and network-region sensitivity analysis and structured channel pruning were combined to reduce redundant computation while retaining fine-grained behavior information. Using a dataset collected from a pig farm in Hunan Province, China, and comprising 2713 images and 30,695 annotated instances after training-set augmentation, we systematically evaluated detection performance and computational complexity through module ablation, pruning-sensitivity analysis, comparisons with mainstream models, class-wise evaluation, and interpretability analysis. The proposed model provides a lightweight visual solution for automated behavior monitoring of group-housed pigs.

## 2. Materials and Methods

### 2.1. Dataset

#### 2.1.1. Farm Environment and Data Sources

The dataset was collected from a large-scale pig farm in Hunan Province, China, covering group-housed scenarios in multiple pig pens. The original video recordings were collected during two periods: November–December 2023 and July–September 2024. Images were extracted from video clips recorded in different pens, and the study used static images with their corresponding target annotations. The pens differed in pig distribution, individual postures, occlusion conditions, and background layouts, providing multi-scenario samples for fine-grained pig behavior recognition. The videos covered routine daily activities, from which representative behavior frames were selected. The dataset was designed to retain variation in target density, occlusion level, posture, and local illumination conditions as much as possible, thereby reducing the model’s dependence on a single scene pattern.

#### 2.1.2. Behavioral Categories and Data Labeling

Based on pig posture, feeding/drinking status, and functional-facility targets, 12 detection classes were defined, including 10 fine-grained pig behavior classes and two functional-facility classes, feeder and drinker. The behavior classes were formed by combining posture and interaction states to describe feeding, drinking, and idle behaviors under standing, sitting, and lying postures (side_lying, sitting_drinking, sitting_eating, sitting_idle, standing_drinking, standing_eating, standing_idle, lying_drinking, lying_eating, and lying_idle). Behavioral annotation was based on the combined assessment of body posture, mouth position, head orientation, and visible interaction cues. Specifically, standing_eating, sitting_eating, and lying_eating represented standing, sitting, and lying postures, respectively, with the mouth positioned at the feeder access area, the head oriented toward the feed, and observable cues consistent with feeding interaction. Similarly, standing_drinking, sitting_drinking, and lying_drinking represented the corresponding postures with the mouth positioned at the drinker and visible cues indicating drinking-related interaction. Proximity to or contact with a feeder or drinker alone was not considered sufficient to assign an eating or drinking label. Standing_idle, sitting_idle, and lying_idle indicated the corresponding postures without visible feeding- or drinking-related interactions, with lying_idle specifically restricted to sternal recumbency. In contrast, side_lying represented lateral recumbency, characterized by the lateral side of the torso resting against the floor, and was distinguished from lying_idle by body orientation and floor contact. The feeder and drinker classes referred to the corresponding functional facilities. The operational definitions and observable criteria for the 10 fine-grained pig behavior categories are presented in Table 1.

**Figure 1:**
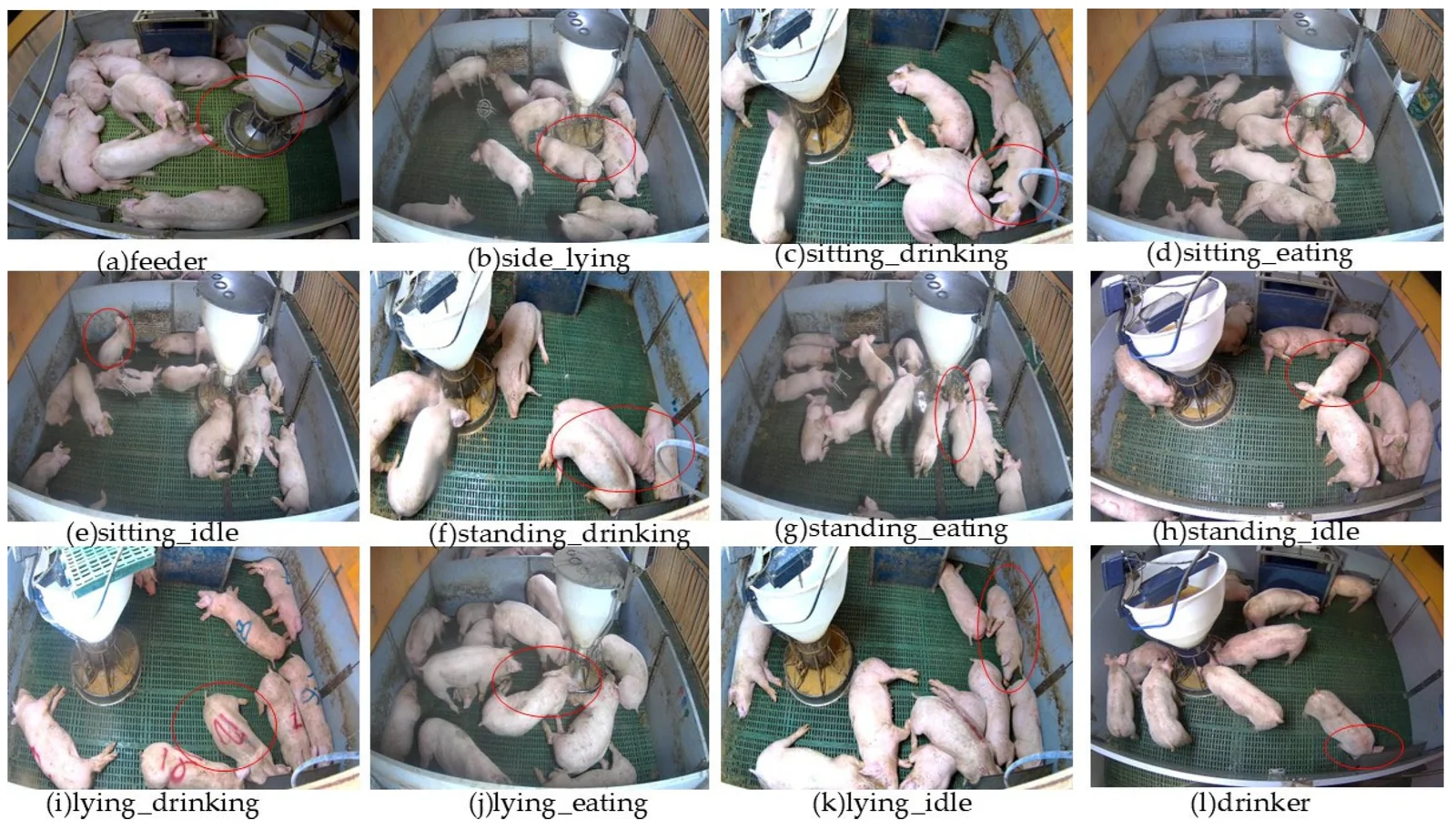
Examples of 10 behavioral categories and 2 functional facilities. The red circles indicate typical examples of the corresponding labels. (**a**) feeder; (**b**) side_lying; (**c**) sitting_drinking; (**d**) sitting_eating; (**e**) sitting_idle; (**f**) standing_drinking; (**g**) standing_eating; (**h**) standing_idle; (**i**) lying_drinking; (**j**) lying_eating; (**k**) lying_idle; (**l**) drinker.

All images were annotated on an instance-by-instance basis using rectangular bounding boxes. Occluded targets were annotated when their classes and locations could still be reliably determined from visible appearance cues, whereas completely unidentifiable targets were not annotated. Examples of the 10 behavioral categories and two functional facilities are shown in Figure 1.

#### 2.1.3. Dataset Partitioning

Following video frame extraction at intervals of 15 frames using a Python script (version 3.8.20), the extracted frames were saved in JPG format and manually screened to remove blurred and low-quality images. The retained images were subsequently annotated using LabelImg (version: 1.8.6, https://github.com/HumanSignal/labelImg, accessed on 1 April 2026).

The dataset comprised 21 source videos recorded from 20 distinct pig pens across two data collection periods, with 10 different pig pens included in each period. To reduce potential information leakage between data subsets, the dataset was partitioned according to the source pig pens. Specifically, eleven source videos from eleven pig pens were allocated to the training set, five source videos from five other pig pens to the validation set, and five source videos from the remaining four pig pens to the test set. The pig pens assigned to the three subsets were mutually exclusive, ensuring that videos and images originating from the same pig pen were not distributed across different subsets.

**Figure 2:**
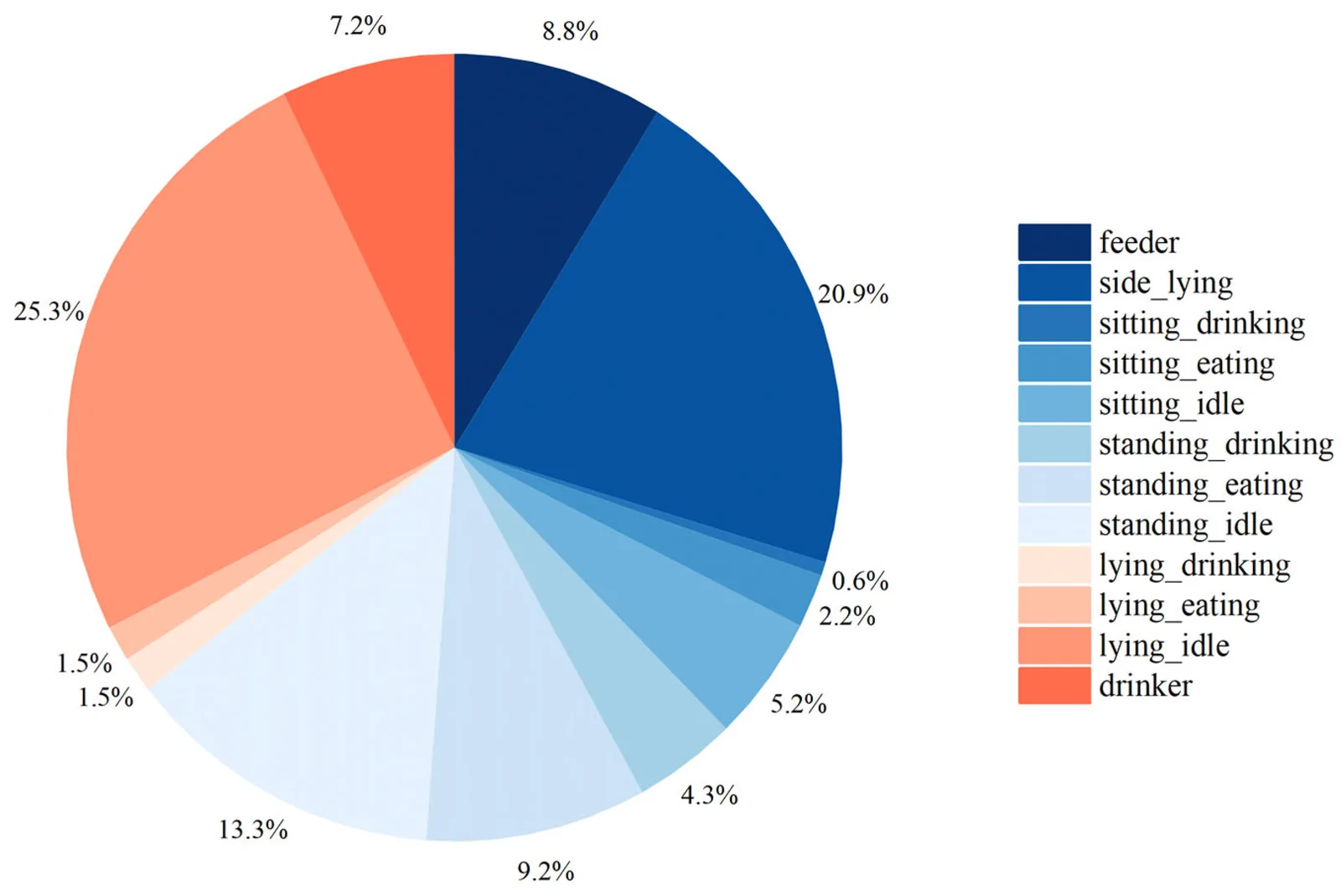
Instance proportions of the behavioral and functional-facility categories in the dataset.

Offline data augmentation was applied exclusively to the training set after partitioning to increase sample diversity. After data augmentation, the final dataset comprised 2713 images and 30,695 annotated instances, including 2170 training images with 24,546 instances, 406 validation images with 4605 instances, and 137 test images with 1544 instances. The resulting image counts corresponded to an approximate ratio of 16:3:1 across the training, validation, and test sets. This ratio reflects the final image counts after training-set augmentation rather than the proportions of the original images before augmentation. All models used the same dataset partition for training and evaluation, and the test set was used only for final performance evaluation. The instance proportions of the behavioral and functional-facility categories are shown in Figure 2.

### 2.2. EPP-YOLOv8n Method

#### 2.2.1. YOLOv8n Baseline Model

**Figure 3:**
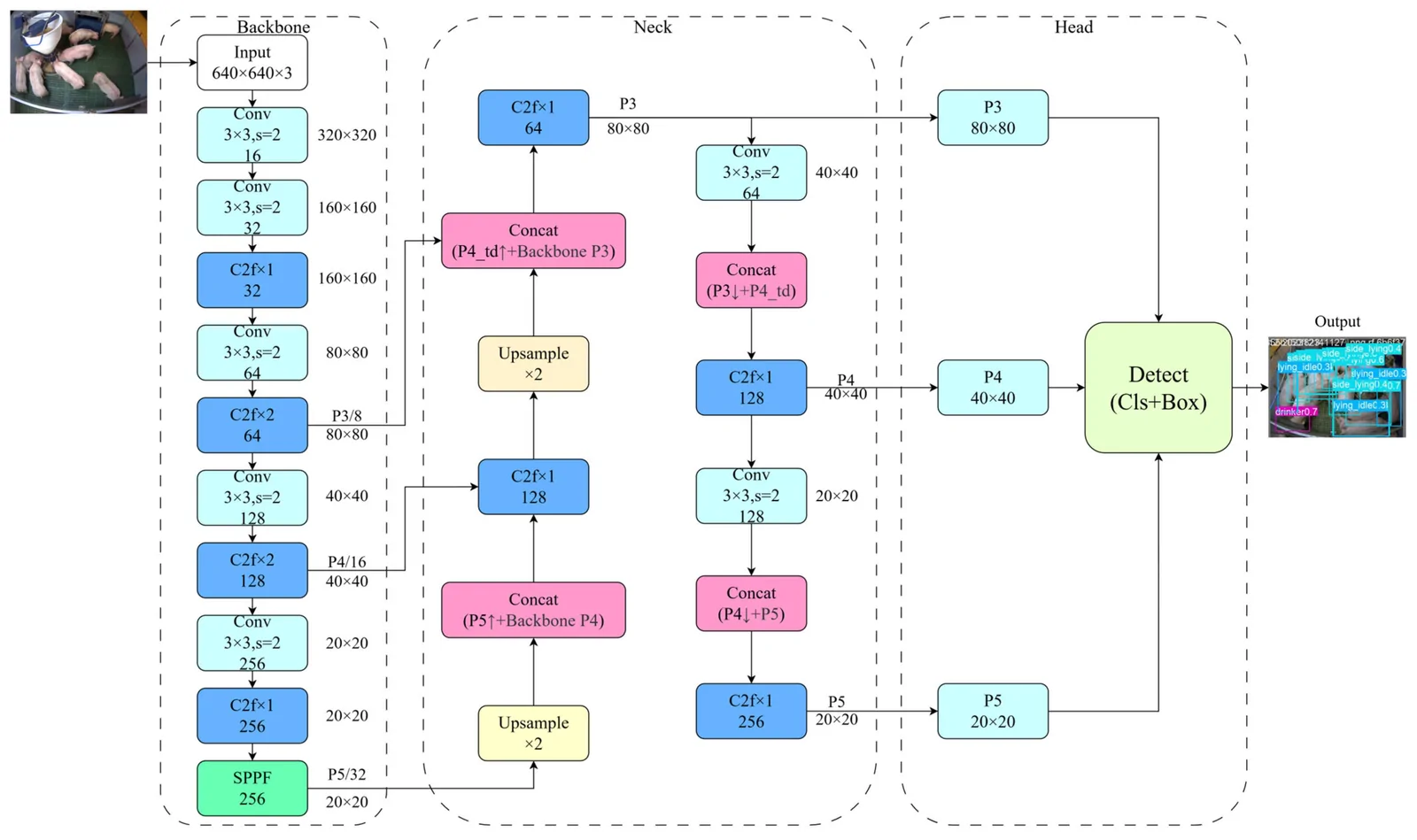
YOLOv8n network architecture.

This study used YOLOv8n released by Ultralytics as the baseline model [22]. YOLOv8n is a single-stage, anchor-free object detector consisting of a Backbone, Neck, and Head. The Backbone extracts multi-scale features through convolutional layers, C2f modules, and the spatial pyramid pooling-fast (SPPF) module. The Neck combines the top-down feature transmission of the Feature Pyramid Network (FPN) [23] with the bottom-up feature aggregation of Path Aggregation Network (PANet) [24] to enable multi-scale information exchange. The Head uses a decoupled detection design and combines Distribution Focal Loss (DFL) [25] with a task-aligned positive-sample assignment strategy [26] for object detection. Owing to its compact architecture and relatively low parameter count, YOLOv8n was selected as the baseline model. The network architecture is shown in Figure 3.

#### 2.2.2. Overall Structure of EPP-YOLOv8n

**Figure 4:**
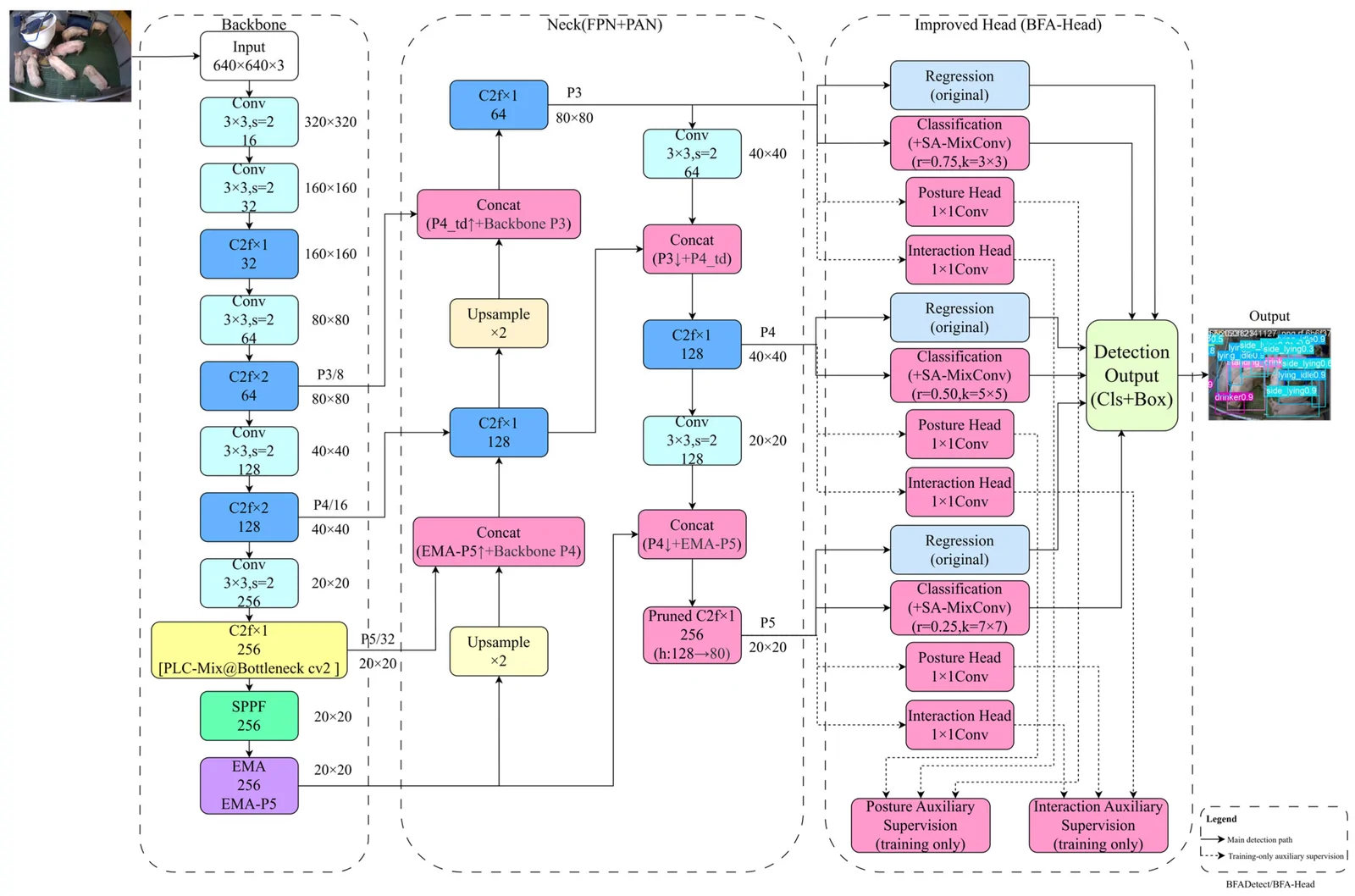
Overall architecture of EPP-YOLOv8n.

To balance fine-grained behavior recognition accuracy and model complexity in group-housed pigs, this study developed EPP-YOLOv8n (Enhanced Perception and Pruning YOLOv8n) based on YOLOv8n, as shown in Figure 4.

The model contains four main improvements. First, EMA is inserted after the SPPF module in the Backbone to enhance high-level semantic feature representation. Second, BFA-Head is designed to combine scale-adaptive feature extraction with posture- and interaction-assisted supervision to guide fine-grained behavior feature learning. Third, PLC-Mix is selectively introduced into the Bottleneck.cv2 component of the P5 C2f module (Backbone layer 8) to reduce redundant computation while preserving the original P3 and P4 feature pathways. Finally, structured channel pruning is applied to the Neck P5 Bottom-up path based on pruning-sensitivity analysis, followed by recovery training to compensate for performance loss caused by structural compression.

#### 2.2.3. Efficient Multi-Scale Attention (EMA)

In group-housed pig pens, mutual occlusion among pigs, posture variation, and complex backgrounds can interfere with the extraction of behavior-related features, particularly local information around the head, torso, and interactions with functional facilities. Attention mechanisms can adaptively adjust feature responses to emphasize informative regions. Ouyang et al. [27] proposed Efficient Multi-Scale Attention (EMA), which integrates multi-scale contextual information through grouped feature processing and cross-spatial information interaction, thereby enhancing feature representation with limited additional computational cost.

**Figure 5:**
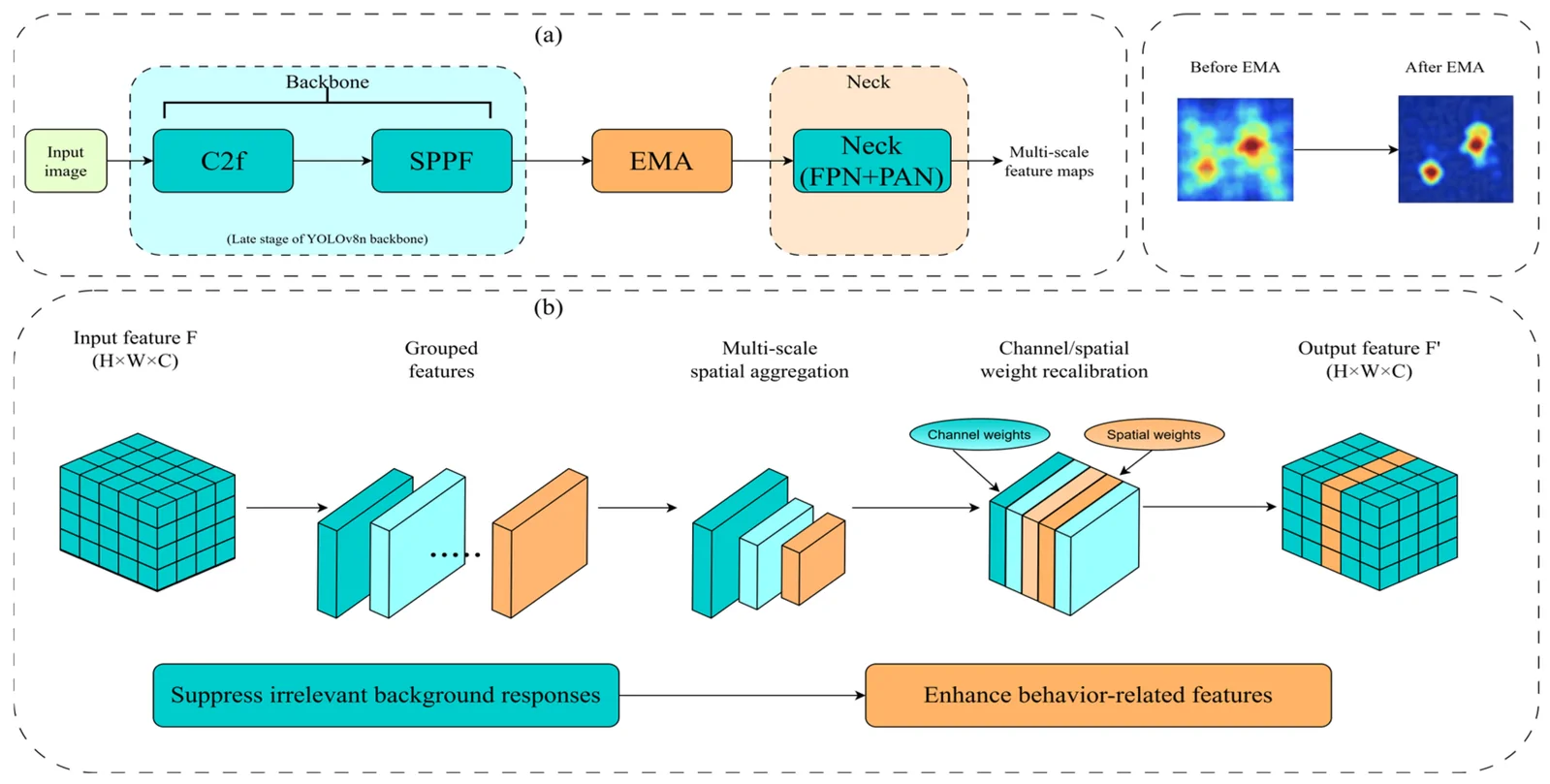
EMA insertion position and functional mechanism. (**a**) Position of EMA in YOLOv8n; (**b**) conceptual mechanism of EMA.

To improve the extraction of fine-grained behavior-related features, this study inserted the EMA module after the SPPF module in the YOLOv8n backbone, as shown in Figure 5. SPPF aggregates contextual information through multi-scale pooling and therefore provides rich high-level semantic features. EMA further recalibrates the responses at different spatial locations, enabling the network to make better use of discriminative information from overall pig posture and local interaction regions. The enhanced high-level features are then fed into the Neck for multi-scale fusion, improving behavior feature representation in complex group-housed environments.

#### 2.2.4. Behavior-Factorized Asymmetric Head (BFA-Head)

Fine-grained composite pig behaviors are jointly determined by overall posture and local interaction state. Relying only on global appearance makes it difficult to distinguish behaviors with similar postures or identical interaction attributes. Inspired by the multi-task feature-sharing concept [28], this study designed the Behavior-Factorized Asymmetric Head (BFA-Head). The head optimizes the classification-branch structure and uses posture- and interaction-assisted supervision to guide the learning of fine-grained behavior features.

In terms of network architecture, BFA-Head retains the original YOLOv8n bounding-box regression branch and replaces the second feature-extraction module in the P3, P4, and P5 classification branches with a scale-adaptive mixed convolution module (SA-MixConv). Tan and Le [29] proposed MixConv, which combines depthwise convolution kernels of different sizes to extract features with different receptive fields and provides a basis for multi-scale convolution design. Inspired by this approach, the channel-processing ratios of P3, P4, and P5 were set to 0.75, 0.50, and 0.25, respectively, with corresponding depthwise convolution kernel sizes of 3 × 3, 5 × 5, and 7 × 7. P3 emphasizes high-resolution local details, P4 captures both local interaction features and overall posture information, and P5 focuses on high-level semantics and contextual features. This design gives the classification branches scale-specific feature extraction capability while keeping the bounding-box regression path unchanged.

To further strengthen behavior-related feature learning, two auxiliary supervision labels were generated online from the original 12 detection classes according to their posture and interaction attributes, without additional manual annotation. The posture factors include standing, sitting, lying, and side_lying, whereas the interaction factors include drinking, eating, and idle. The feeder and drinker facility classes do not participate in either auxiliary task, while side_lying participates only in the posture auxiliary task. The correspondence between the original detection classes and auxiliary labels is shown in Table 2.

The generation of the auxiliary labels follows the original target-assignment process of YOLOv8. Specifically, TaskAlignedAssigner first identifies foreground samples matched to ground-truth targets, after which the original 12-class ground-truth (GT) category IDs are recovered from the matching results. Let *c_j_* denote the original detection class of the *j*-th matched positive sample. Its posture and interaction labels are then generated online using fixed mapping functions *M_p_*(·) and *M_i_*(·), respectively.

(1) $$y_{j}^{p}=M_{p}\left(c_{j}\right)$$

(2) $$y_{j}^{p}=M_{i}\left(c_{j}\right)$$

where *y_j_^p^* and *y_j_^i^* denote the posture and interaction labels corresponding to the *j*-th positive sample, respectively. When the mapping result is −1, the sample does not participate in the loss calculation for the corresponding auxiliary task. This process directly reuses the positive-sample assignment results of TaskAlignedAssigner in the main detection task and therefore does not require changes to the original dataset label files.

The posture- and interaction-assisted branches are both supervised using cross-entropy loss. Let *Ω_p_* and *Ω_i_* denote the sets of valid posture and interaction samples, respectively. The posture auxiliary loss can be expressed as:

(3) $$L_{\mathrm{posture}}=-\frac{1}{\left|\Omega_{P}\right|}\sum_{\mathrm{j\epsilon}\Omega_{P}}\log p_{j,y_{j}^{p}}^{p}$$

The interaction auxiliary loss is expressed as:

(4) $$L_{\mathrm{interaction}}=-\frac{1}{\left|\Omega_{i}\right|}\sum_{\mathrm{j\epsilon}\Omega_{i}}\log p_{j,y_{j}^{i}}^{i}$$

where $p_{j,y_{j}^{p}}^{p}$ and $p_{j,y_{j}^{i}}^{i}$ denote the predicted probabilities of the *j*-th valid positive sample for the true posture and interaction categories, respectively. In the implementation, the corresponding cross-entropy loss is calculated only for positive samples with mapped labels greater than or equal to 0.

In the final model, the posture and interaction auxiliary losses were both assigned a weight of 0.15 (*λ_p_* and *λ_i_*), whereas the consistency-loss weight was set to 0. Therefore, the total training loss of BFA-Head is expressed as:

*L_BAF_* = *L_det_* + *λ_p_L_posture_* + *λ_i_L_Sinteraction_* (5)

*L_det_* the original YOLOv8 detection loss consists of bounding-box regression loss, classification loss, and DFL (Distribution Focal Loss). No additional consistency loss is introduced in the configuration. In the implementation, the two auxiliary losses are weighted and added to the standard detection loss according to their respective weights.

**Figure 6:**
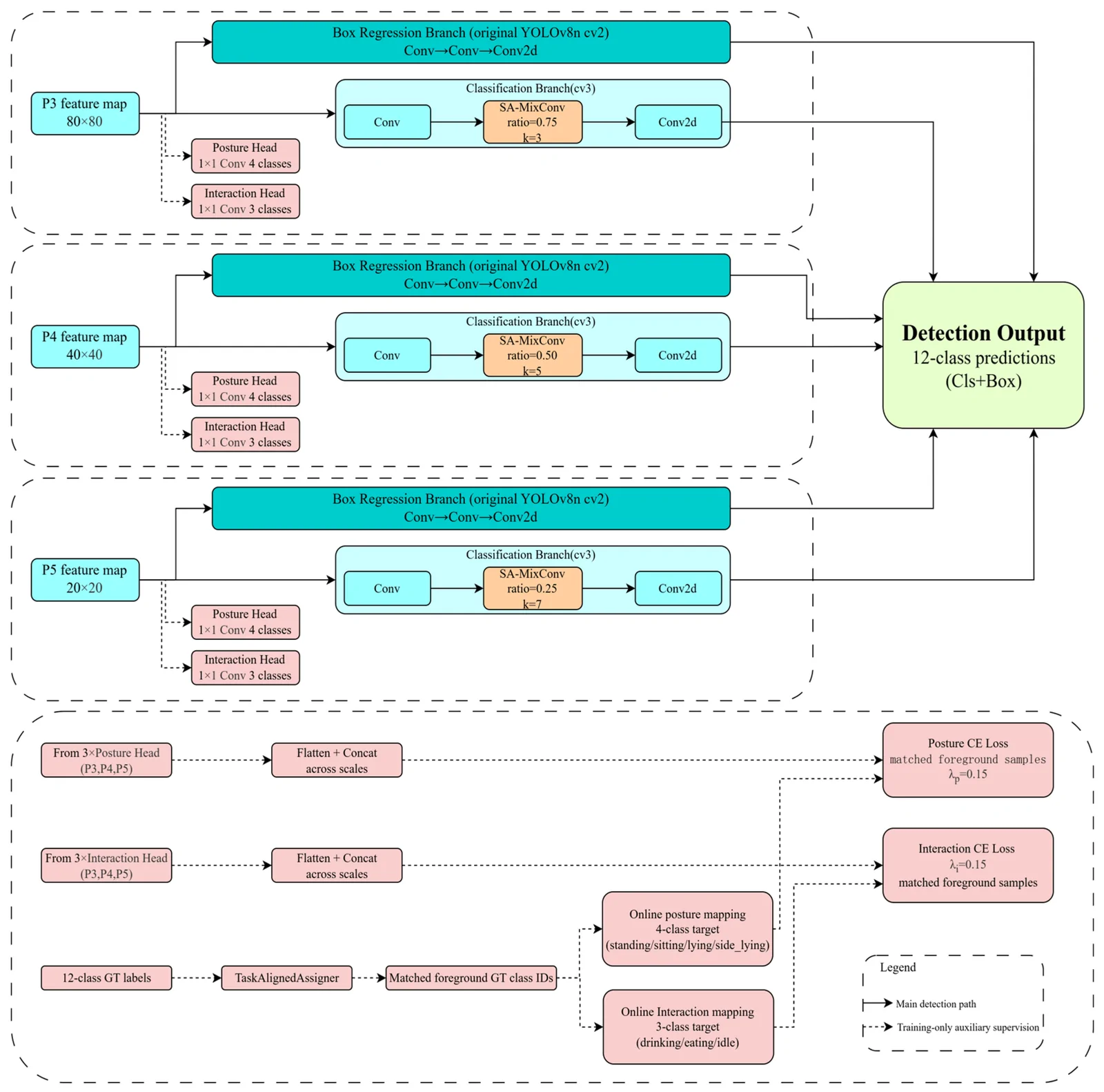
BFA-Head and its auxiliary supervision mechanism.

The posture- and interaction-assisted branches are used only during training to constrain the learning of shared features. During validation and inference, BFA-Head directly returns the standard detection outputs without executing the auxiliary prediction paths. Before deployment, the posture and interaction auxiliary classifiers can also be physically removed from the network. Therefore, the auxiliary supervision does not change the final 12-class detection output format or introduce additional auxiliary prediction paths during deployment. The BFA-Head and its auxiliary supervision mechanism are shown in Figure 6.

#### 2.2.5. P5-Selective PLC-Mix

**Figure 7:**
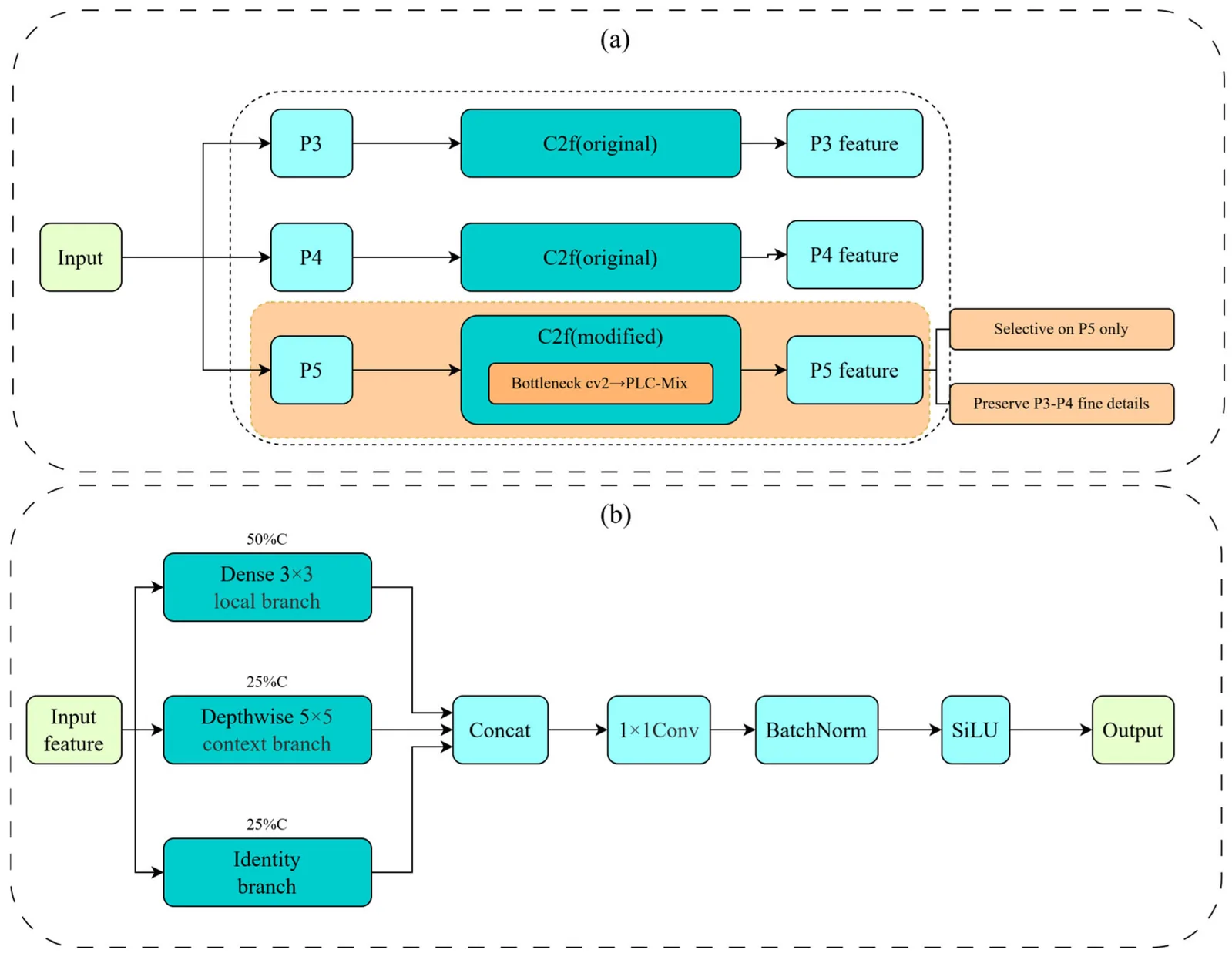
Structure and deployment position of P5-selective PLC-Mix. (**a**) Position of PLC-Mix in YOLOv8n; (**b**) internal structure of PLC-Mix.

Different feature scales play different roles in pig behavior recognition: P3 and P4 mainly preserve local spatial details, whereas P5 emphasizes high-level semantic and contextual information. Inspired by lightweight computation and multi-scale feature modeling [11,13], this study designed P5-selective PLC-Mix and deployed it within the Bottleneck.cv2 component of the P5 C2f module in the Backbone (layer 8), while retaining the original P3 and P4 structures. PLC-Mix combines partial-channel computation with multi-branch feature recombination. Specifically, the input channels are divided into three branches: the first branch contains 50% of the input channels and uses a dense 3 × 3 convolution to extract local spatial features; the second contains 25% of the input channels and uses a 5 × 5 depthwise convolution (DW) to enlarge the spatial receptive field and enhance contextual information; the third contains the remaining 25% and uses an identity mapping to retain the original features without redundant convolution over all channels. The outputs of the three branches are concatenated along the channel dimension and then passed through a 1 × 1 convolution, batch normalization (BN), and SiLU activation to complete feature fusion and recombination. The module structure and deployment position of P5-selective PLC-Mix are shown in Figure 7.

#### 2.2.6. Task-Aware Structured Pruning and Recovery Training

Although lightweight network structures can reduce redundant computation, different feature layers contribute differently to fine-grained pig behavior recognition, so further structural compression must balance model complexity and detection performance. Structured channel pruning removes redundant channels to produce a regular network structure and is an important approach for reducing parameter count and computational cost. Luo et al. [12] combined model pruning with knowledge distillation to develop YOLOv8-Piglet and demonstrated the potential of model compression techniques for pig behavior detection. Based on this work, this study adopted a task-aware structured pruning strategy, using network-region sensitivity analysis to determine suitable pruning locations and recovery training to mitigate the performance loss caused by structural compression.

Different feature scales and fusion paths may contribute differently to fine-grained behavior information. Therefore, pruning sensitivity was first analyzed for six candidate network regions: the C2f modules in Backbone P3, Backbone P4, Neck P4 Top-down, Neck P3 Top-down, Neck P4 Bottom-up, and Neck P5 Bottom-up. To avoid changing feature transfer between modules, the external input and output channel numbers of the target modules were kept unchanged, and only the internal hidden channels of C2f were compressed. A local hidden-channel compression ratio of 12.5% was applied to each candidate region, corresponding to channel changes of 32 → 28, 64 → 56, and 128 → 112. Each candidate model was independently generated from the same unpruned parent model, with only one region compressed at a time; no cumulative pruning or recovery training was performed. Recall, mAP@0.5:0.95, and changes in model complexity were compared on the same validation set to assess the sensitivity of different network regions to structural compression.

After the pruning region was determined, performance under different compression intensities was further analyzed. Network Slimming proposed by Liu et al. [30] uses the scaling factors of batch-normalization layers to evaluate channel importance and provides a basis for structured channel pruning. Inspired by this approach, the magnitude of grouped BN scaling factors was used to evaluate channel importance. The BN scaling factors corresponding to the same hidden-channel index in the C2f module were jointly evaluated, and hidden channels with lower importance were preferentially pruned. In the Neck P5 Bottom-up path, the hidden channels of the target C2f module were successively reduced from 128 to 112, 104, 96, 88, 80, and 72, corresponding to local compression ratios of 12.5%, 18.75%, 25.0%, 31.25%, 37.5%, and 43.75%, respectively, to examine the effects of different compression levels on detection performance and model complexity.

**Figure 8:**
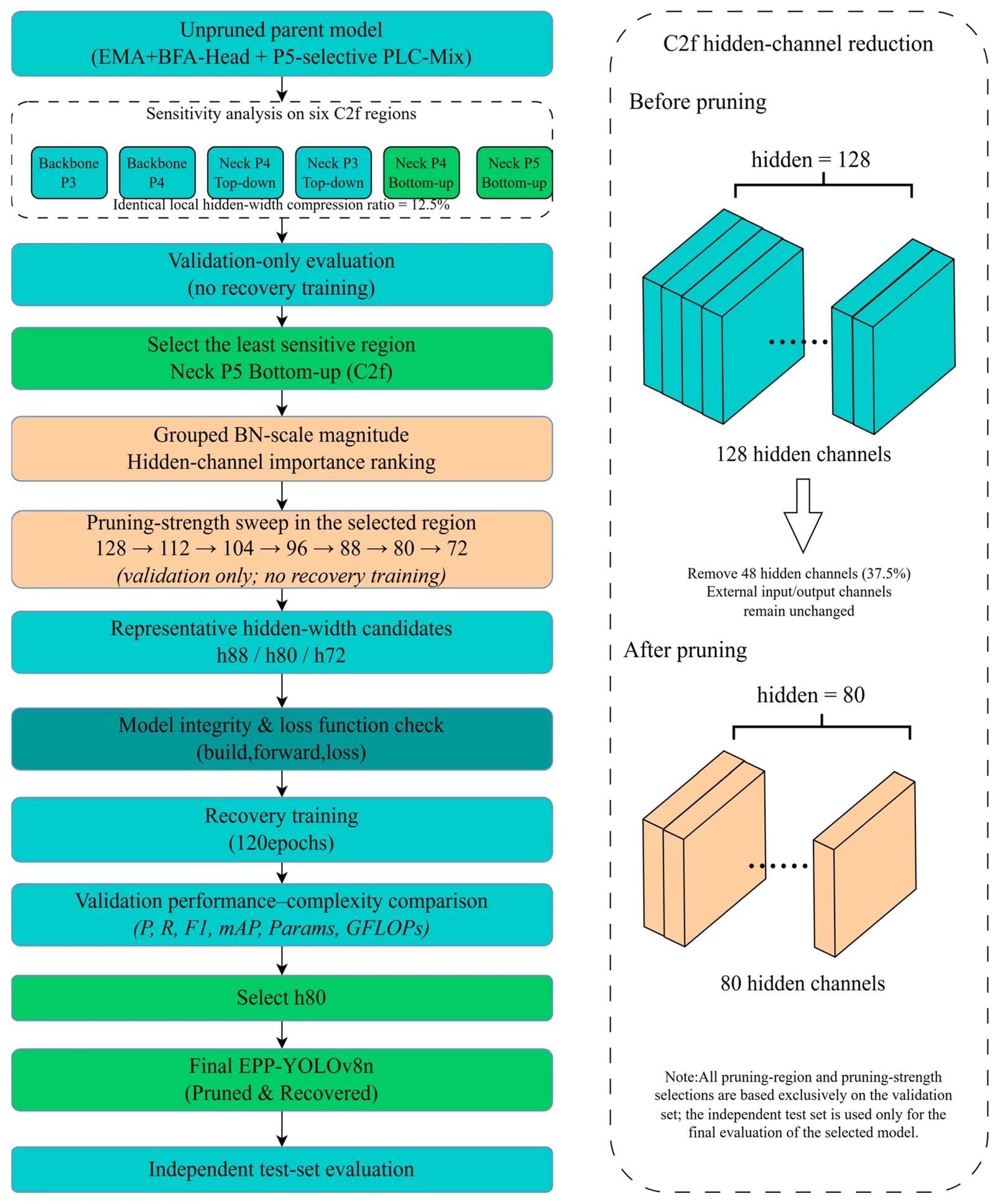
Task-aware structured pruning and recovery training process.

Because channel pruning without recovery training may reduce performance, three representative structures with 88, 80, and 72 hidden channels were selected for 120 epochs of recovery training. Under the same training settings, the recovered candidates were compared in terms of detection performance, parameter count, and computational complexity, and the final pruning configuration was determined from the validation results. The C2f module in the Neck P5 Bottom-up path was ultimately selected for formal pruning, with its hidden channels reduced from 128 to 80, corresponding to a reduction of 48 hidden channels and a local compression ratio of 37.5%. This region selection, channel compression, and recovery-training procedure further reduces model complexity while retaining fine-grained behavior-discriminative information as much as possible. The task-aware structured pruning and recovery training process is shown in Figure 8.

## 3. Results

### 3.1. Experimental Environment and Training Setup

Model training and performance evaluation were conducted on Windows 11 using an NVIDIA GeForce RTX 3060 Laptop GPU with 6 GB of memory. The deep learning framework was PyTorch 2.4.1+cu118, and the object detection models were implemented with Ultralytics 8.4.67. To ensure comparability, all models used the same dataset partition and followed the same main training and evaluation procedures.

The input image size was 640 × 640, and the batch size was 16. The AdamW optimizer proposed by Loshchilov and Hutter [31] was used, with an initial learning rate of 0.002, a final learning-rate factor of 0.01, momentum of 0.9, weight decay of 0.0005, and 3 warmup epochs. The random seed was fixed at 42. Deterministic computation and automatic mixed precision (AMP) training were enabled to reduce the influence of stochastic factors on the experimental results.

To ensure comparability, all models used the same dataset partition and a unified evaluation protocol, and the standard models were trained for 120 epochs. In the structured pruning experiments, the selected pruning candidates underwent an additional 120 epochs of recovery training to compensate for performance loss caused by channel compression. All models were evaluated using the same validation and test data. For a training-budget-matched comparison, the unpruned model incorporating EMA, BFA-Head, and P5-selective PLC-Mix was further trained for 120 epochs under the same optimization settings used for the pruned model’s recovery training.

### 3.2. Evaluation Metrics

To comprehensively evaluate the proposed model for fine-grained pig behavior detection, performance was assessed in terms of detection accuracy and model complexity. Precision (P), recall (R), mAP@0.5, and mAP@0.5:0.95 were used to evaluate detection performance, while parameter count (Params) and floating-point operations (GFLOPs) were used to evaluate model complexity.

Precision represents the proportion of correctly detected targets among all predicted targets, whereas recall represents the proportion of ground-truth targets correctly detected by the model. For behavioral categories with limited test instances, 95% confidence intervals for recall were calculated using the Wilson score method based on the number of correctly detected instances and the total number of ground-truth instances in each category. The overall precision and recall values reported in this study were calculated as the arithmetic means of the corresponding class-wise metrics across the 12 detection classes at the selected confidence threshold.

Average precision (AP) was calculated from the precision–recall curve following the COCO evaluation protocol [32], using 101 uniformly distributed recall points. mAP@0.5 represents the mean AP across all 12 detection classes at an intersection over union (IoU) threshold of 0.50, whereas mAP@0.5:0.95 averages AP across IoU thresholds from 0.50 to 0.95 in increments of 0.05. The mAP metrics were calculated using COCOeval in pycocotools, with low-confidence predictions retained to preserve the complete confidence ranking. The corresponding mathematical definitions are provided in Appendix A.

Params represents the total number of learnable parameters, reflecting model size. GFLOPs represents the floating-point operations required for one forward pass with an input image size of 640 × 640, reflecting theoretical computational complexity. Lower Params and GFLOPs indicate a smaller model and reduced theoretical computational requirements, respectively.

### 3.3. Ablation Study of Architectural Improvements

To systematically evaluate the individual and combined contributions of the proposed architectural improvements, a complete ablation study was conducted using three modules: EMA, BFA-Head, and P5-selective PLC-Mix. Eight configurations (E0–E7) were established to cover all possible combinations of these modules. This full-factorial design enabled the individual effects of each module and their combined effects to be evaluated under consistent experimental conditions. All configurations were trained for 120 epochs under the same experimental settings, without structured pruning or recovery training. The results are presented in Table 3.

Compared with the baseline E0, introducing EMA alone (E1) increased mAP@0.5:0.95 from 87.43% to 88.48%, with negligible changes in model complexity. Introducing BFA-Head alone (E2) reduced the parameter count and GFLOPs by 3.08% and 6.57%, respectively, while increasing precision by 0.89 percentage points, although mAP@0.5:0.95 decreased by 0.47 percentage points. The standalone PLC-Mix configuration (E3) reduced the parameter count by 3.10% and GFLOPs by 0.91%, while maintaining comparable detection accuracy. The combined configurations exhibited different performance trade-offs. The EMA + BFA-Head configuration (E4) achieved higher precision and lower computational complexity than E1, whereas EMA + PLC-Mix (E5) maintained an mAP@0.5:0.95 of 88.14% with reduced computational requirements. Combining BFA-Head and PLC-Mix (E6) further reduced the parameter count to 2.827 M and GFLOPs to 7.592. The complete three-module configuration (E7) achieved the highest recall (96.74%) and mAP@0.5 (98.66%) among the eight configurations, with an mAP@0.5:0.95 of 88.11%. Relative to the baseline, E7 improved recall and mAP@0.5:0.95 by 3.76 and 0.68 percentage points, respectively, while reducing the parameter count and GFLOPs by 6.16% and 7.22%. Although E1 achieved the highest mAP@0.5:0.95, the complete configuration provided a favorable balance between recall, detection accuracy, and computational complexity. These results indicate that the individual modules contribute differently to the overall performance, and their combination involves trade-offs rather than uniform improvements across all evaluation metrics.

### 3.4. Comparison with Other Object Detection Models

To further evaluate the proposed architectural improvements before structured pruning on the fine-grained pig behavior detection task, several representative object detection models were selected for comparison. SSD300-VGG16 is a classic single-stage detector [33], whereas Faster R-CNN-R50-FPN is a representative two-stage detector [34]. YOLOv5n [35], YOLOv7-tiny [36], and YOLOX-Nano [37] were selected as lightweight YOLO-series models, and the Transformer-based real-time detector RT-DETR-R18 [38] was also included. The original YOLOv8n [22] was used as the baseline to evaluate the effectiveness of the proposed improvements. The unpruned three-module configuration (E7), trained for 120 epochs, was used for comparison to distinguish architectural improvements from subsequent model compression. All models used the same dataset partition, and mAP was calculated according to the unified COCO evaluation protocol. Precision and recall were evaluated on the test set using the confidence thresholds determined for each model from the validation set. The performance and complexity comparison is presented in Table 4.

As shown in Table 4, the unpruned E7 model achieved precision, recall, mAP@0.5, and mAP@0.5:0.95 values of 96.40%, 96.74%, 98.66%, and 88.11%, respectively. Its recall, mAP@0.5, and mAP@0.5:0.95 were higher than those of all comparison models. Compared with the original YOLOv8n, E7 improved recall by 3.76 percentage points and mAP@0.5:0.95 by 0.68 percentage points.

In terms of model complexity, E7 contained 2.828 M parameters and required 7.613 GFLOPs, representing reductions of approximately 6.16% and 7.22%, respectively, compared with YOLOv8n. Although YOLOv5n and YOLOX-Nano had smaller model sizes, their mAP@0.5:0.95 values were lower than that of E7. These results demonstrate that the proposed architectural improvements achieved a favorable balance between detection performance and computational complexity before structured pruning. The additional compression achieved by structured pruning was evaluated separately.

### 3.5. Structured Pruning and Training-Budget-Matched Evaluation

Table 5 shows that different network regions exhibit significantly different sensitivities to the same proportion of structured channel compression. Specifically, after compressing 12.5% of the hidden channels, the mAP@0.5:0.95 of Backbone P3 decreased from 86.59% to 40.66%, indicating that this region is highly sensitive to structural perturbation without recovery training. In contrast, the mAP@0.5:0.95 of Neck P5 Bottom-up decreased by only 2.35 percentage points, indicating better performance retention without recovery. Therefore, Neck P5 Bottom-up was selected for subsequent pruning-intensity search. After the pruning location was determined, different hidden widths were evaluated on the Neck P5 Bottom-up path, as shown in Table 6. As hidden width decreased from 128 to 72, parameter count and computational cost decreased continuously, while detection performance without recovery training generally declined. When hidden width was reduced from 88 to 80, mAP@0.5:0.95 decreased from 77.88% to 69.99%, indicating a marked increase in structural perturbation. Therefore, performance without recovery training alone could not determine the final structure, and recovery ability at different compression levels needed to be further evaluated.

Based on the above results, three representative structures with 88, 80, and 72 hidden channels were selected for 120 epochs of recovery training. The validation results are shown in Table 7. After recovery training, h80 achieved an mAP@0.5:0.95 of 88.64%, higher than h88 (88.52%) and h72 (88.27%). Its recall reached 95.35%, also higher than the other two candidate structures. Compared with h88, h80 further reduced parameter count and GFLOPs without decreasing mAP@0.5:0.95. Although h72 further reduced model size, its recall and mAP@0.5:0.95 were lower than those of h80. Therefore, considering both detection performance and model complexity, the final pruning configuration used a hidden width of 80 in the Neck P5 Bottom-up path.

To distinguish the contribution of structured pruning from the effect of additional training, a training-budget-matched control experiment was conducted. Starting from the same 120-epoch-trained E7 parent model, E7-Extended underwent an additional 120 epochs of training without pruning, using optimization settings matched to those employed during the recovery training of E7-Pruned. Both models were evaluated using the same protocol, including validation-set-based confidence threshold selection and COCO evaluation on the same test set. The comparative results are presented in Table 8.

As shown in Table 8, E7-Extended achieved an mAP@0.5:0.95 of 89.29%, compared with 88.11% for the original E7 model and 89.07% for E7-Pruned. Under the matched training budget, E7-Pruned reduced the parameter count by 8.98% and GFLOPs by 2.67% relative to E7-Extended, with a decrease of 0.22 percentage points in mAP@0.5:0.95. These results indicate that additional training contributed to the observed accuracy improvement, while structured pruning primarily reduced model complexity with a relatively small loss in detection accuracy.

### 3.6. Final Test Performance and Class-Wise Results

To further evaluate the overall detection performance of EPP-YOLOv8n, model evaluation was conducted on a test set containing 137 images and 1544 annotated instances. The optimal confidence threshold of 0.774 was determined from the validation-set macro-F1 score and then fixed for calculating precision and recall on the test set. The mAP metric was obtained using the unified COCO evaluation protocol. The comparison between EPP-YOLOv8n and the original YOLOv8n is presented in Table 9.

As shown in Table 9, YOLOv8n achieved precision, recall, mAP@0.5, and mAP@0.5:0.95 values of 96.08%, 92.98%, 97.50%, and 87.43%, respectively. In comparison, EPP-YOLOv8n achieved 98.13%, 94.50%, 98.28%, and 89.07%, corresponding to increases of 2.05, 1.52, 0.78, and 1.64 percentage points, respectively. The parameter count decreased from 3.013 M to 2.574 M, and GFLOPs decreased from 8.206 to 7.410, corresponding to reductions of 14.59% and 9.70%, respectively. These results indicate that EPP-YOLOv8n improved overall detection performance on the current test set while reducing model complexity.

As shown in Table 10, the class-wise results further show that EPP-YOLOv8n achieved high performance for several categories. For feeding behavior, both precision and recall reached 100.00% for sitting_eating and lying_eating. For functional facilities, feeder and drinker achieved precision values of 99.24% and 99.08%, respectively, indicating reliable identification of the feeder and drinker targets in the test set.

In contrast, some categories with similar postures or less distinctive local interaction features remained more difficult to recognize. The recall and AP@0.5:0.95 of lying_drinking were 85.19% and 82.17%, respectively, making it one of the weaker categories in the current test set. The AP@0.5:0.95 values of standing_idle and sitting_idle were 83.52% and 88.33%, respectively. These results indicate that some idle behaviors and lying_drinking remain challenging, likely because their overall postures are similar and local head position or interactions with functional facilities may be affected by occlusion.

The number of instances differed among classes in the test set. In particular, sitting_drinking and lying_eating contained only 15 and 20 instances, respectively. Their recall values were 93.33% and 100.00%, corresponding to 95% Wilson score confidence intervals of 70.18–98.81% and 83.89–100.00%, respectively. The relatively wide intervals reflect the small sample sizes of these categories.

Overall, EPP-YOLOv8n achieved good overall detection performance on the current test set, with strong results for several behavior categories, while reducing parameter count and computational complexity.

### 3.7. Confusion Matrix and Error-Type Analysis

Based on the results recorded by the official DetectionValidator, a detection confusion matrix was constructed with the confidence threshold fixed at 0.774 and the IoU threshold fixed at 0.50. Among the 1544 ground-truth instances, 1483 targets were successfully matched with predictions, including 1461 correct class predictions and 22 class confusions. Another 61 ground-truth targets remained unmatched, and only three unmatched false-positive predictions were recorded. Overall, the main errors consisted of unmatched ground-truth targets and a small number of class confusions among fine-grained behavior categories.

**Figure 9:**
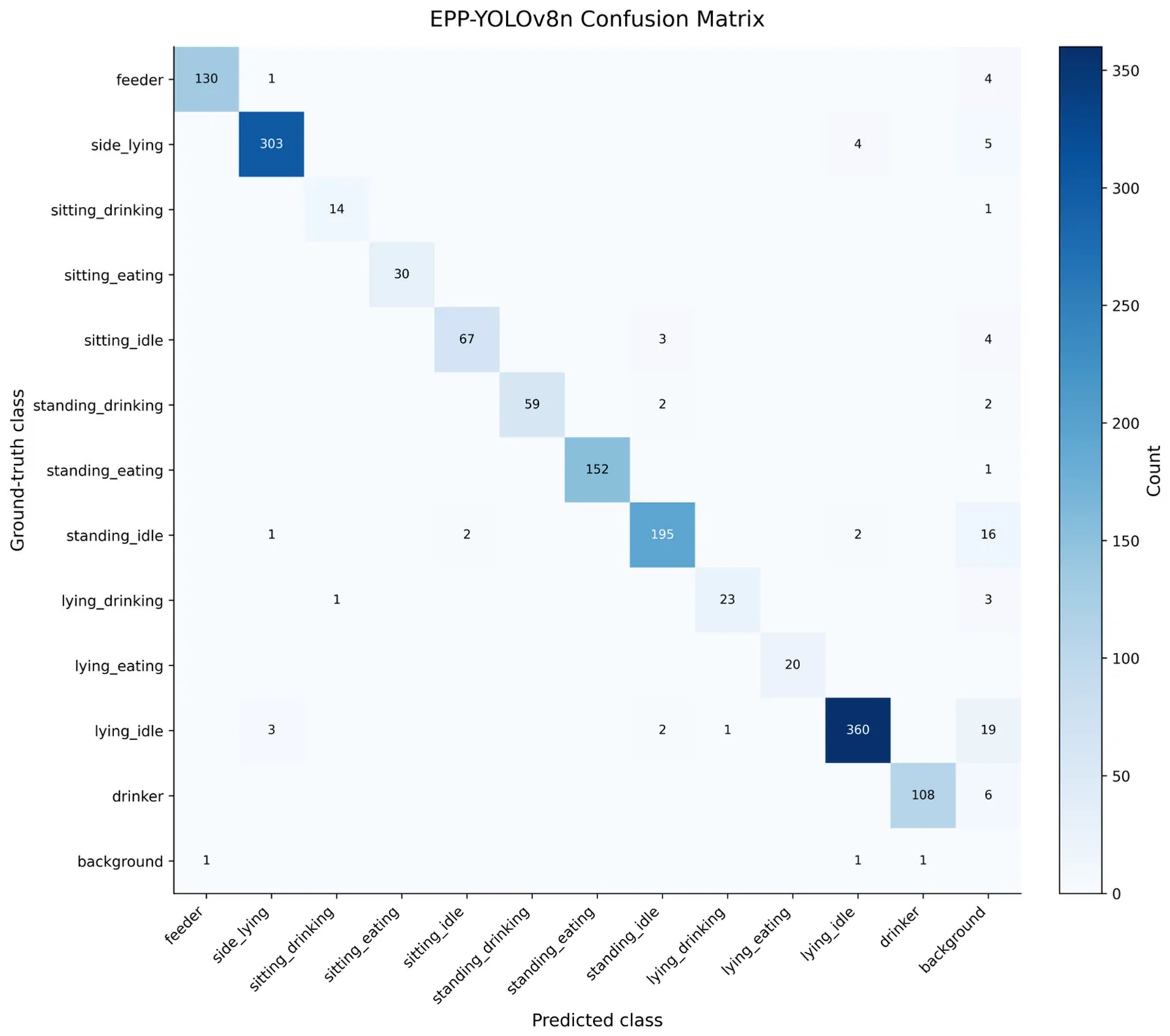
Confusion matrix of EPP-YOLOv8n on the test set.

Further analysis showed that the main class confusions were side_lying→lying_idle (4 cases, 1.28% of the ground-truth side_lying instances), sitting_idle→standing_idle (3 cases, 4.05%), lying_idle→side_lying (3 cases, 0.78%), and standing_drinking→standing_idle (2 cases, 3.17%). The unmatched rates of lying_drinking, standing_idle, sitting_drinking, and sitting_idle were 11.11%, 7.41%, 6.67%, and 5.41%, respectively. These rates represent the proportions of ground-truth instances that were not matched to any prediction box at the specified confidence and IoU thresholds, excluding instances matched to predictions with incorrect class labels. These results indicate that recognition remains difficult for some fine-grained behaviors with similar postures or weakly differentiated interaction states. Combined with the qualitative detection results, local occlusion and insufficient visual information in interaction regions may further increase the difficulty of recognizing these behaviors. The EPP-YOLOv8n confusion matrix on the test set is shown in Figure 9.

### 3.8. Visual Comparison of Detection Results in Representative Scenes

**Figure 10:**
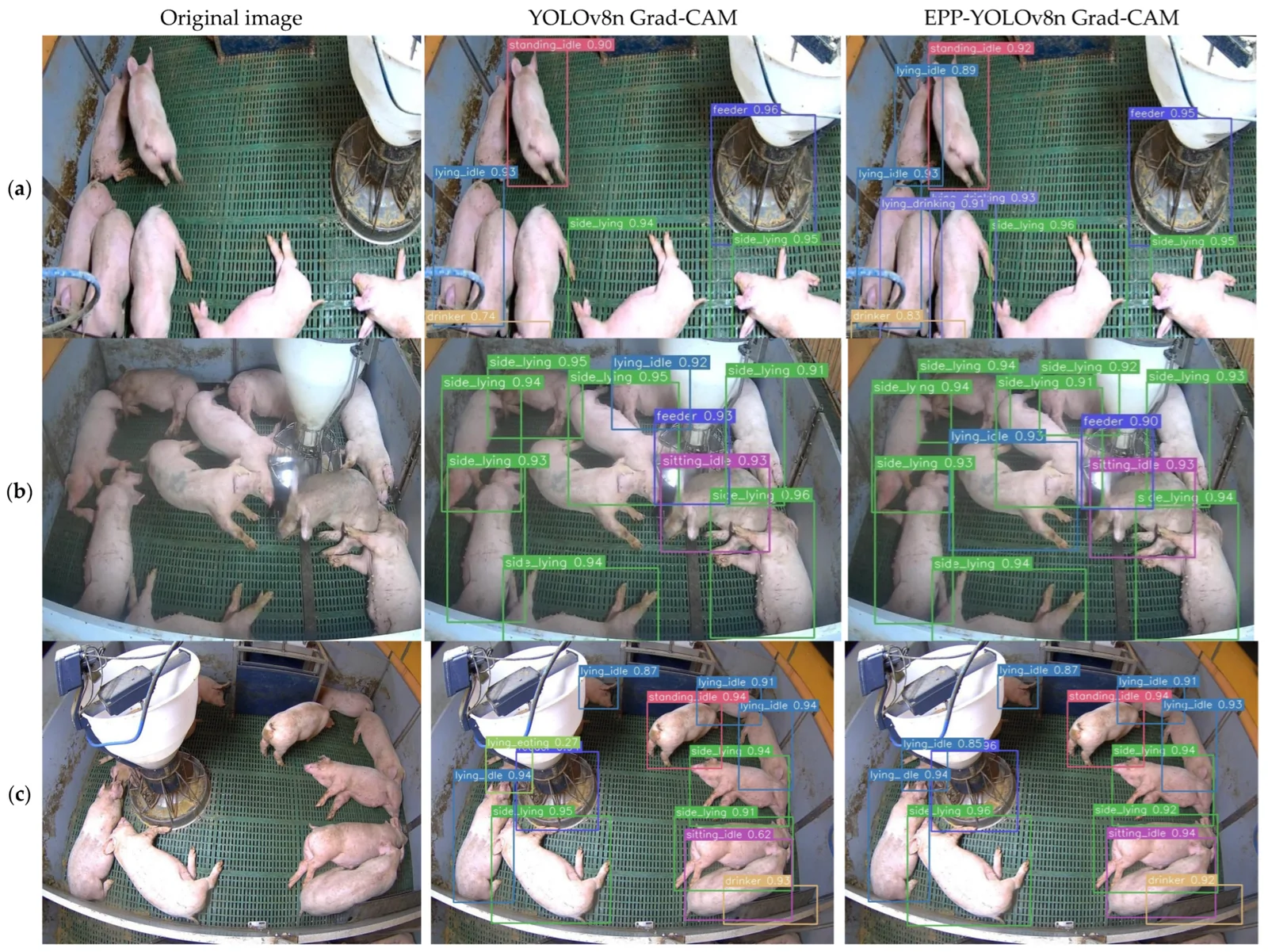
Comparison of YOLOv8n and EPP-YOLOv8n detection results in representative complex scenes. (**a**) Scene containing different postures and local interaction behaviors; (**b**) scene with multiple pigs clustered and relatively obvious individual occlusion; (**c**) scene involving a pig lying near the feeder, where feeding and resting behaviors are easily confused.

To qualitatively evaluate the detection performance of EPP-YOLOv8n under different farming conditions, representative detection results of YOLOv8n and EPP-YOLOv8n are compared in Figure 10. Figure 10a presents a scene involving multiple postures and local behavioral interactions, while Figure 10b illustrates a crowded scene with substantial overlap and occlusion among individual pigs. These scenes provide a visual comparison of the two models in complex group-housing environments. Figure 10c further illustrates the challenge of distinguishing visually similar fine-grained behaviors. In this scene, a pig was lying close to the feeder without engaging in feeding behavior. The baseline YOLOv8n produced an additional low-confidence prediction of lying_eating (0.27), indicating potential confusion between feeding and resting behaviors. In contrast, EPP-YOLOv8n predicted lying_idle (0.85) in the corresponding region, consistent with the observed behavior. This result suggests that the improved model can reduce behavior confusion associated with spatial proximity to feeding equipment in this example.

Overall, the qualitative comparisons complement the quantitative evaluation by illustrating the detection characteristics of the two models across different postures, crowded conditions, and visually similar behaviors.

### 3.9. Instance-Level Grad-CAM Interpretability Analysis

**Figure 11:**
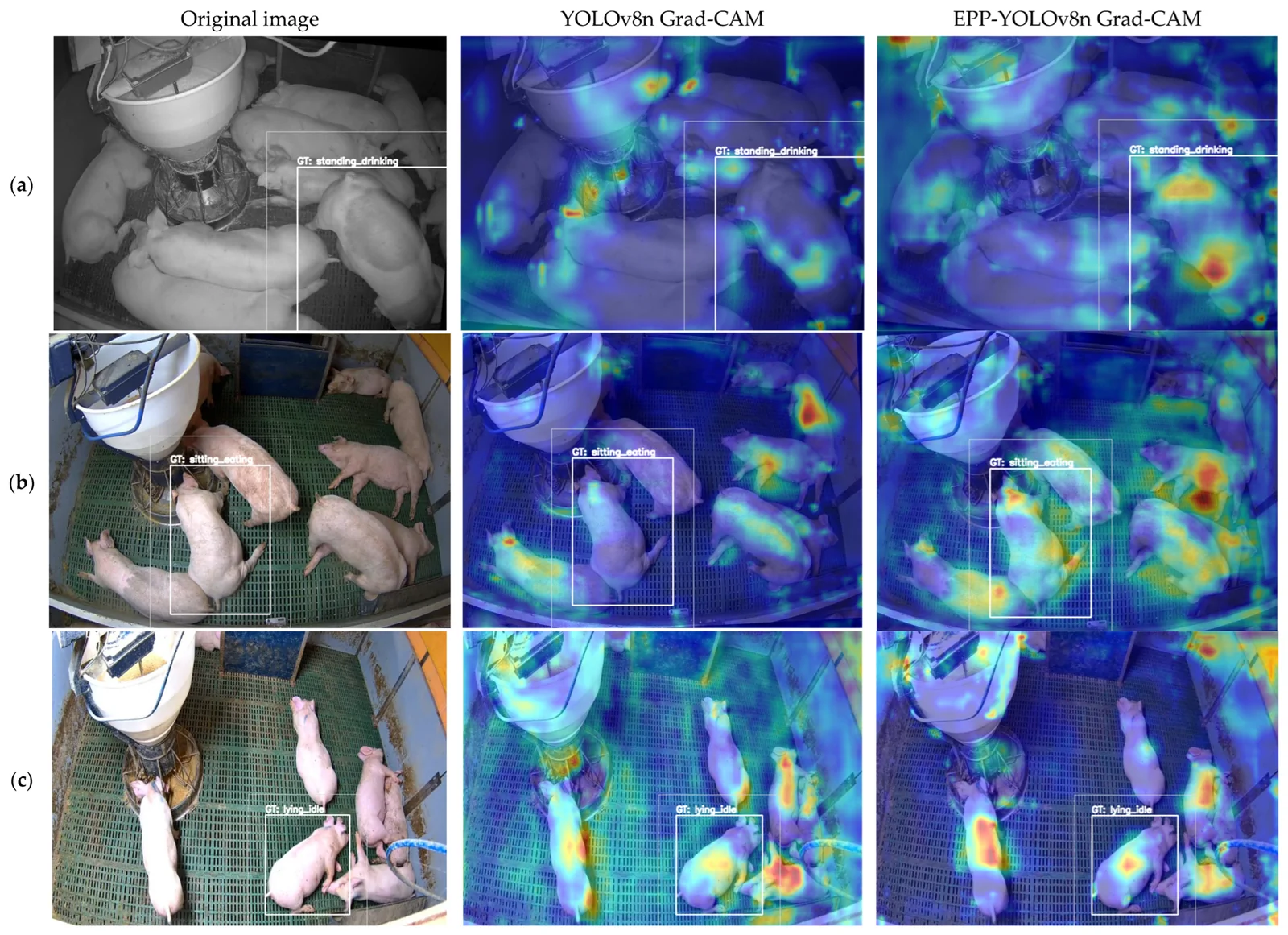
Comparison of instance-level Grad-CAM visualizations between YOLOv8n and EPP-YOLOv8n for representative fine-grained pig behaviors. (**a**) standing_drinking; (**b**) sitting_eating; (**c**) lying_idle. Each group presents the original image, YOLOv8n Grad-CAM, and EPP-YOLOv8n Grad-CAM from left to right. White bounding boxes indicate the selected ground-truth instances, while the outer boxes indicate the expanded contextual regions.

To further investigate the feature response characteristics of EPP-YOLOv8n, instance-level Grad-CAM [39] visualizations were generated and compared with those of the baseline YOLOv8n, as shown in Figure 11. Three representative behaviors were selected, including standing_drinking, sitting_eating, and lying_idle. For each example, the same ground-truth instance was used to guide the visualization of both models. Grad-CAM responses from the P3, P4, and P5 feature levels were fused to examine the spatial distribution of features associated with the target instance.

As shown in Figure 11a, for the standing_drinking behavior, the baseline model exhibits scattered responses across the surrounding pigs and feeding equipment, whereas EPP-YOLOv8n shows a greater proportion of responses within the target region. A similar tendency is observed for the sitting_eating behavior in Figure 11b, where the improved model displays more visible responses around the target pig’s head and body. In Figure 11c, the two models exhibit different response patterns for lying_idle, with EPP-YOLOv8n showing more localized responses in certain regions while retaining activations associated with neighboring pigs.

## 4. Discussion

This study improved YOLOv8n using EMA, BFA-Head, P5-selective PLC-Mix, and structured pruning to enhance fine-grained pig behavior feature representation while reducing model complexity. EPP-YOLOv8n achieved an mAP@0.5:0.95 of 89.07%, an increase of 1.64 percentage points over YOLOv8n, while parameter count and GFLOPs decreased by 14.59% and 9.70%, respectively, indicating a favorable balance between detection performance and model complexity on the current test set. The improved modules contributed differently to the final model. EMA enhanced feature representation, whereas BFA-Head primarily reduced computational cost in the classification branches and guided behavior feature learning through posture- and interaction-assisted supervision. The ablation results showed that adding BFA-Head to the EMA-equipped configuration reduced the parameter count and GFLOPs by 3.09% and 6.55%, respectively, and increased precision by 0.72 percentage points, while recall and mAP@0.5:0.95 decreased by 2.77 and 0.62 percentage points, respectively. These results demonstrate the lightweighting effectiveness of BFA-Head and the associated trade-off in detection performance. The subsequent introduction of PLC-Mix and structured pruning further reduced model complexity while preserving competitive detection performance. Pruning-sensitivity analysis indicated that the Neck P5 Bottom-up path was relatively insensitive to channel compression, and selective compression followed by recovery training further reduced redundant computation.

The class-wise results showed that lying_drinking and some idle behaviors remained relatively difficult to recognize. The confusion matrix revealed confusion between side_lying and lying_idle and between sitting_idle and standing_idle. These behaviors have similar overall postures, and their differences are mainly reflected in local body appearance or interactions with functional facilities; occlusion and insufficient local visual information may further increase recognition difficulty. The Grad-CAM visualizations revealed differences in feature-response distribution between the two models, with more localized target-region responses in selected standing_drinking and sitting_eating examples.

## 5. Conclusions

To address individual occlusion in group-housed pig pens, limited visual differences among fine-grained behaviors, and the need to control model computational complexity, this study proposed EPP-YOLOv8n, a fine-grained pig behavior recognition method based on improved YOLOv8n. First, the efficient multi-scale attention module (EMA) was inserted after the SPPF module in the backbone to enhance high-level semantic feature representation. Second, BFA-Head was designed with SA-MixConv in the classification branches and posture- and interaction-assisted supervision to strengthen learning of discriminative information for fine-grained composite behaviors. Third, P5-selective PLC-Mix was introduced into the Backbone P5 C2f module to reduce redundant computation while preserving high-level semantic feature representation. Finally, structured channel pruning was performed based on network-region sensitivity analysis, reducing the hidden channels of the C2f module in Neck P5 Bottom-up from 128 to 80, followed by 120 epochs of recovery training to obtain the final model.

Experiments were conducted on a dataset containing 2713 images and 30,695 annotated instances. EPP-YOLOv8n achieved precision, recall, mAP@0.5, and mAP@0.5:0.95 values of 98.13%, 94.50%, 98.28%, and 89.07%, respectively, on the test set. Compared with the original YOLOv8n, these metrics increased by 2.05, 1.52, 0.78, and 1.64 percentage points, respectively. The parameter count decreased from 3.013 M to 2.574 M, and GFLOPs decreased from 8.206 to 7.410, corresponding to reductions of 14.59% and 9.70%, respectively. The ablation and pruning-sensitivity analyses further showed that the improved modules had different effects on detection performance and model complexity, and the final model achieved a favorable balance between the two on the current test set.

This study has several limitations. First, the data were collected mainly from a single pig farm, and the generalizability of the proposed model to other farms and housing conditions has not yet been fully validated. Second, some behavioral categories contained relatively few test instances, which may affect the reliability of their performance estimates. Future work will expand data collection across multiple farms and evaluate the model using independently collected data under different housing and illumination conditions. Continuous video-based behavior analysis and temporal feature modeling will also be explored to improve the discrimination of visually similar behaviors. In addition, deployment on edge devices will be investigated, with emphasis on inference speed, resource consumption, and operational stability in practical farming environments.

## Figures and Tables

**Table 1:** Operational definitions and observable criteria for fine-grained pig behavior categories.

Behavior Category	Postural Characteristics	Operational Definition and Observable Criteria
side_lying	Lateral recumbency, with one side of the torso resting against the floor.	Identified by lateral body orientation and contact between the side of the torso and the floor, rather than the sternal recumbency characteristic of lying_idle.
sitting_drinking	Sitting posture, with the hindquarters resting on the floor.	The mouth is positioned at the drinker outlet, with head orientation and visible interaction cues consistent with drinking.
sitting_eating	Sitting posture, with the hindquarters resting on the floor and the forelimbs supporting the anterior body.	The mouth is positioned at the feeder access area, with head orientation and visible interaction cues consistent with feeding.
sitting_idle	Sitting posture, with the hindquarters resting on the floor.	No observable feeding- or drinking-related interaction is present.
standing_drinking	Standing posture, with the body supported by all four limbs.	The mouth is positioned at the drinker outlet, with head orientation and visible interaction cues consistent with drinking.
standing_eating	Standing posture, with the body supported by all four limbs.	The mouth is positioned at the feeder access area, with the head oriented toward the feed and visible cues consistent with feeding interaction.
standing_idle	Standing posture, with the body supported by all four limbs.	No observable feeding- or drinking-related interaction is present.
lying_drinking	Recumbent posture, with the body resting on the floor.	The mouth is positioned at the drinker outlet, with head orientation and visible interaction cues consistent with drinking.
lying_eating	Recumbent posture, with the body resting on the floor.	The mouth is positioned at the feeder access area, with head orientation and visible interaction cues consistent with feeding.
lying_idle	Sternal recumbency, with the ventral surface of the body resting on the floor.	No observable feeding- or drinking-related interaction is present. Distinguished from side_lying by torso orientation and floor contact.

Note: Eating and drinking behaviors were identified through the combined assessment of mouth position, head orientation, and visible interaction cues. Proximity to or contact with a feeder or drinker alone was insufficient for assigning the corresponding behavior labels.

**Table 2:** Correspondence between original detection classes and auxiliary labels.

Original Detection Class	Posture Label	Interaction Label
feeder	ignore	ignore
side_lying	side_lying	ignore
sitting_drinking	sitting	drinking
sitting_eating	sitting	eating
sitting_idle	sitting	idle
standing_drinking	standing	drinking
standing_eating	standing	eating
standing_idle	standing	idle
lying_drinking	lying	drinking
lying_eating	lying	eating
lying_idle	lying	idle
drinker	ignore	ignore

**Table 3:** Ablation results of individual and combined architectural improvements in EPP-YOLOv8n.

Model	EMA	BFA-Head	P5-Selective PLC-Mix	P (%)	R (%)	mAP@0.5 (%)	mAP@0.5:0.95 (%)	Params (M)	GFLOPs
E0	–	–	–	96.08	92.98	97.50	87.43	3.013	8.206
E1	✓	–	–	96.52	93.96	98.33	88.48	3.014	8.227
E2	–	✓	–	96.97	93.36	97.12	86.96	2.920	7.667
E3	–	–	✓	96.28	93.88	97.71	87.50	2.920	8.131
E4	✓	✓	–	97.24	91.19	97.97	87.86	2.921	7.688
E5	✓	–	✓	96.25	92.98	97.82	88.14	2.920	8.152
E6	–	✓	✓	97.22	94.27	98.19	87.80	2.827	7.592
E7	✓	✓	✓	96.40	96.74	98.66	88.11	2.828	7.613

Note: “✓” indicates the inclusion of the corresponding module, whereas “–” indicates its absence. All eight configurations were evaluated without structured pruning. P, precision; R, recall; mAP, mean average precision; Params, parameter count; GFLOPs, billions of floating-point operations.

**Table 4:** Performance and complexity comparison of different object detection models.

Model	Input Size	P (%)	R (%)	mAP@0.5 (%)	mAP@0.5:0.95 (%)	Params (M)	GFLOPs
SSD	300	86.67	81.71	87.43	66.17	25.216	61.951
Faster R-CNN	640	91.68	86.88	93.20	76.81	41.409	182.262
YOLOv5n	640	86.98	83.17	90.16	74.87	1.775	4.177
YOLOv7-tiny	640	94.50	89.07	94.13	78.19	6.037	13.117
YOLOX-Nano	640	96.17	92.75	96.28	79.69	0.899	2.560
RT-DETR	640	91.23	85.81	94.17	84.61	20.097	60.461
YOLOv8n	640	96.08	92.98	97.50	87.43	3.013	8.206
E7 (Unpruned)	640	96.40	96.74	98.66	88.11	2.828	7.613

**Table 5:** Structured pruning sensitivity results for different candidate regions at the same compression rate.

Pruning Region	Layer	Hidden Channels	Local Compression (%)	Parameter Reduction (%)	GFLOPs Reduction (%)	R (%)	mAP@0.5:0.95 (%)
Unpruned Parent	—	—	0.00	0.000	0.000	94.47	86.59
Backbone P3	4	32 → 28	12.50	0.362	1.729	59.59	40.66
Backbone P4	6	64 → 56	12.50	1.445	1.721	74.32	69.74
Neck P4 Top-down	12	64 → 56	12.50	0.941	1.120	88.77	75.74
Neck P3 Top-down	15	32 → 28	12.50	0.236	1.126	90.50	68.86
Neck P4 Bottom-up	18	64 → 56	12.50	0.832	0.991	90.30	77.44
Neck P5 Bottom-up	21	128 → 112	12.50	3.322	0.988	92.31	84.24

Note: The evaluation results in the table are from the validation set results, not the final test set results. The same applies to Table 6 and Table 7.

**Table 6:** Validation results without recovery training for the Neck P5 Bottom-up path at different pruning intensities.

Hidden Channels	Local Compression (%)	Parameter Reduction (%)	GFLOPs Reduction (%)	P (%)	R (%)	mAP@0.5 (%)	mAP@0.5:0.95 (%)
128	0.00	0.000	0.000	95.51	94.47	97.18	86.59
112	12.50	3.322	0.988	96.53	92.31	96.98	84.24
104	18.75	4.861	1.446	95.25	92.35	96.69	83.25
96	25.00	6.319	1.880	94.19	92.57	96.27	80.15
88	31.25	7.694	2.289	94.10	90.31	95.72	77.88
80	37.50	8.988	2.674	83.96	85.18	91.73	69.99
72	43.75	10.201	3.035	81.13	74.76	86.96	66.44

**Table 7:** Validation results of representative pruning intensities after 120 epochs of recovery training.

Hidden Channels	Compression (%)	Params (M)	GFLOPs	P (%)	R (%)	mAP@0.5 (%)	mAP@0.5:0.95 (%)
88	31.25	2.610	7.439	95.99	95.06	97.82	88.52
80	37.50	2.574	7.410	95.98	95.35	97.98	88.64
72	43.75	2.539	7.382	96.41	94.83	97.74	88.27

**Table 8:** Performance comparison of pruned and unpruned models under matched training budgets.

Model	Epochs	P (%)	R (%)	mAP@0.5 (%)	mAP@0.5:0.95 (%)	Params (M)	GFLOPs
E7	120	96.40	96.74	98.66	88.11	2.828	7.613
E7-Extended	120 + 120	98.01	95.90	98.58	89.29	2.828	7.613
E7-Pruned	120 + 120	98.13	94.50	98.28	89.07	2.574	7.410

Note: E7 represents the unpruned model incorporating EMA, BFA-Head, and PLC-Mix. E7-Extended denotes the same model trained for an additional 120 epochs without pruning, whereas E7-Pruned denotes its structured-pruned counterpart after 120 epochs of recovery training.

**Table 9:** Final test results of YOLOv8n and EPP-YOLOv8n.

Model	P (%)	R (%)	mAP@0.5 (%)	mAP@0.5:0.95 (%)	Params (M)	GFLOPs
YOLOv8n	96.08	92.98	97.50	87.43	3.013	8.206
EPP-YOLOv8n	98.13	94.50	98.28	89.07	2.574	7.410

**Table 10:** Class-wise test performance of EPP-YOLOv8n.

Class	Instances	P (%)	R (%)	AP@0.5 (%)	AP@0.5:0.95 (%)
feeder	135	99.24	96.30	98.93	93.42
side_lying	312	98.38	97.12	98.64	89.61
sitting_drinking	15	93.33	93.33	98.76	89.52
sitting_eating	30	100.00	100.00	100.00	93.82
sitting_idle	74	97.10	90.54	98.06	88.33
standing_drinking	63	100.00	93.65	97.68	88.28
standing_eating	153	100.00	99.35	100.00	91.42
standing_idle	216	96.53	90.28	96.77	83.52
lying_drinking	27	95.83	85.19	92.93	82.17
lying_eating	20	100.00	100.00	100.00	91.88
lying_idle	385	98.09	93.51	97.93	87.32
drinker	114	99.08	94.74	99.61	89.58

## Data Availability

The data presented in this study are available upon request from the corresponding author. The data are not publicly available due to privacy restrictions.

## Appendix A. Mathematical Definitions of Evaluation Metrics

(A1) $$P=\frac{\mathrm{TP}}{\mathrm{TP}+\mathrm{FP}}$$

(A2) $$R=\frac{\mathrm{TP}}{\mathrm{TP}+\mathrm{FN}}$$

(A3) $$AP_{\tau }^{\left(c\right)}=\frac{1}{101}\sum_{r\in \left\{0,0.01,\ldots ,1.00\right\}}P_{\mathrm{interp},\tau }^{\left(c\right)}\left(r\right)$$

(A4) $$\mathrm{mA}P_{50}=\frac{1}{C}\sum_{c=1}^{C}AP_{0.50}^{\left(c\right)}$$

(A5) $$m{\mathrm{AP}}_{50:95}=\frac{1}{10C}\sum_{\tau \in \left\{0.50,0.55,\ldots ,0.95\right\}}\sum_{c=1}^{C}AP_{\tau }^{\left(c\right)}$$

where *TP*, *FP*, and *FN* represent true positives, false positives, and false negatives, respectively; *C* denotes the total number of detection classes (*C* = 12); *τ* represents the intersection over union (IoU) threshold; *AP_t_*^(*c*)^ denotes the average precision of class *c* at IoU threshold *τ*; and *P*_interp,*τ*^(*c*)^(*r*) represents the interpolated precision of class c at recall level *r* and IoU threshold *τ*.

## References

[1] Rohan A., Rafaq M.S., Hasan M.J., Asghar F., Bashir A.K., Dottorini T. (2024). Application of deep learning for livestock behaviour recognition: A systematic literature review. Comput. Electron. Agric..

[2] Chen C., Zhu W., Norton T. (2021). Behaviour recognition of pigs and cattle: Journey from computer vision to deep learning. Comput. Electron. Agric..

[3] Gómez Y., Stygar A.H., Boumans I.J.M.M., Bokkers E.A.M., Pedersen L.J., Niemi J.K., Pastell M., Manteca X., Llonch P. (2021). A systematic review on validated precision livestock farming technologies for pig production and its potential to assess animal welfare. Front. Vet. Sci..

[4] Chen Z., Lu J., Wang H. (2023). A review of posture detection methods for pigs using deep learning. Appl. Sci..

[5] Riekert M., Opderbeck S., Wild A., Gallmann E. (2021). Model selection for 24/7 pig position and posture detection by 2D camera imaging and deep learning. Comput. Electron. Agric..

[6] Alameer A., Kyriazakis I., Bacardit J. (2020). Automated recognition of postures and drinking behaviour for the detection of compromised health in pigs. Sci. Rep..

[7] Zhuang Y., Zhou K., Zhou Z., Ji H., Teng G. (2023). Systems to monitor the individual feeding and drinking behaviors of growing pigs based on machine vision. Agriculture.

[8] Ji H., Yu J., Lao F., Zhuang Y., Wen Y., Teng G. (2022). Automatic position detection and posture recognition of grouped pigs based on deep learning. Agriculture.

[9] Luo Y., Xia J., Lu H., Luo H., Lv E., Zeng Z., Li B., Meng F., Yang A. (2024). Automatic recognition and quantification feeding behaviors of nursery pigs using improved YOLOV5 and feeding functional area proposals. Animals.

[10] Pu P., Wang J., Yan G., Jiao H., Li H., Lin H. (2025). Enhanced Multi-Scenario Pig Behavior Recognition Based on YOLOv8n. Animals.

[11] Zhang K., Zhang Y., Xu H. (2025). Lightweight domestic pig behavior detection based on YOLOv8. Appl. Sci..

[12] Luo Y., Lin K., Xiao Z., Chen Y., Yang C., Xiao D. (2025). Collaborative optimization of model pruning and knowledge distillation for efficient and lightweight multi-behavior recognition in piglets. Animals.

[13] Zeng Z., Wu Z., Xie R., Lin K., Tan S., He X., Luo Y. (2025). MACA-Net: Mamba-driven adaptive cross-layer attention network for multi-behavior recognition in group-housed pigs. Agriculture.

[14] Wei X.-S., Song Y.-Z., Mac Aodha O., Wu J., Peng Y., Tang J., Yang J., Belongie S. (2022). Fine-grained image analysis with deep learning: A survey. IEEE Trans. Pattern Anal. Mach. Intell..

[15] Hu Y., Dai X., Dai B., Li R., Fang J., Yin Y., Liu H., Shen W. (2025). Feeding behavior recognition of group-housed pigs based on pose estimation and keypoint features discrimination. Comput. Electron. Agric..

[16] Lagua E.B., Mun H.-S., Sharifuzzaman M., Hasan M.K., Mehtab A., Kang J.-G., Park H.-R., Kim Y.-H., Yang C.-J. (2026). Limitations and opportunities of combining postural and nutritive behaviors for comprehensive condition detection in group-housed pigs using YOLO architectures. Smart Agric. Technol..

[17] Wang T., Hu Y., Yin H. (2026). Enhancing pig behavior recognition in complex environments: A transfer learning-assisted YOLO11 network with wavelet convolution and synergistic attention. Animals.

[18] Chen Y., Wu J., Kuang Y., Yang Q. (2026). DWEC-YOLO: A multi-behavior detection model for group-housed pigs. Animals.

[19] Bhujel A., Wang Y., Lu Y., Morris D., Dangol M. (2025). A systematic survey of public computer vision datasets for precision livestock farming. Comput. Electron. Agric..

[20] Li J., Hu X., Lucic A., Wu Y., Condotta I.C.F.S., Dilger R.N., Ahuja N., Green-Miller A.R. (2026). Promote computer vision applications in pig farming scenarios: High-quality dataset, fundamental models, and comparable performance. J. Integr. Agric..

[21] Gu X., Chen J. (2026). Progressive degradation-aware distillation for robust indoor object detection. Sci. Rep..

[22] Jocher G., Chaurasia A., Qiu J. (2023). Ultralytics YOLOv8, version 8.0.0. https://github.com/ultralytics/ultralytics.

[23] Lin T.-Y., Dollár P., Girshick R., He K., Hariharan B., Belongie S. Feature pyramid networks for object detection. Proceedings of the IEEE Conference on Computer Vision and Pattern Recognition (CVPR).

[24] Liu S., Qi L., Qin H., Shi J., Jia J. Path aggregation network for instance segmentation. Proceedings of the IEEE/CVF Conference on Computer Vision and Pattern Recognition (CVPR).

[25] Li X., Wang W., Wu L., Chen S., Hu X., Li J., Tang J., Yang J. (2020). Generalized focal loss: Learning qualified and distributed bounding boxes for dense object detection. Adv. Neural Inf. Process. Syst..

[26] Feng C., Zhong Y., Gao Y., Scott M.R., Huang W. TOOD: Task-aligned one-stage object detection. Proceedings of the IEEE/CVF International Conference on Computer Vision (ICCV).

[27] Ouyang D., He S., Zhang G., Luo M., Guo H., Zhan J., Huang Z. Efficient multi-scale attention module with cross-spatial learning. Proceedings of the IEEE International Conference on Acoustics, Speech and Signal Processing (ICASSP).

[28] Misra I., Shrivastava A., Gupta A., Hebert M. Cross-stitch networks for multi-task learning. Proceedings of the IEEE Conference on Computer Vision and Pattern Recognition (CVPR).

[29] Tan M., Le Q.V. MixConv: Mixed depthwise convolutional kernels. Proceedings of the British Machine Vision Conference (BMVC).

[30] Liu Z., Li J., Shen Z., Huang G., Yan S., Zhang C. Learning efficient convolutional networks through network slimming. Proceedings of the IEEE International Conference on Computer Vision (ICCV).

[31] Loshchilov I., Hutter F. Decoupled weight decay regularization. Proceedings of the International Conference on Learning Representations (ICLR).

[32] Lin T.-Y., Maire M., Belongie S., Hays J., Perona P., Ramanan D., Dollár P., Zitnick C.L. (2014). Microsoft COCO: Common objects in context. Computer Vision—ECCV 2014.

[33] Liu W., Anguelov D., Erhan D., Szegedy C., Reed S., Fu C.-Y., Berg A.C. (2016). SSD: Single shot MultiBox detector. Computer Vision—ECCV 2016.

[34] Ren S., He K., Girshick R., Sun J. (2017). Faster R-CNN: Towards real-time object detection with region proposal networks. IEEE Trans. Pattern Anal. Mach. Intell..

[35] Jocher G., Chaurasia A., Stoken A., Borovec J., Kwon Y., Michael K., Fang J., Yifu Z., Wong C., Montes D. (2022). ultralytics/yolov5: V7.0—YOLOv5 SOTA Realtime Instance Segmentation.

[36] Wang C.-Y., Bochkovskiy A., Liao H.-Y.M. YOLOv7: Trainable bag-of-freebies sets new state-of-the-art for real-time object detectors. Proceedings of the IEEE/CVF Conference on Computer Vision and Pattern Recognition (CVPR).

[37] Ge Z., Liu S., Wang F., Li Z., Sun J. (2021). YOLOX: Exceeding YOLO series in 2021. arXiv.

[38] Zhao Y., Lv W., Xu S., Wei J., Wang G., Dang Q., Liu Y., Chen J. DETRs beat YOLOs on real-time object detection. Proceedings of the IEEE/CVF Conference on Computer Vision and Pattern Recognition (CVPR).

[39] Selvaraju R.R., Cogswell M., Das A., Vedantam R., Parikh D., Batra D. Grad-CAM: Visual explanations from deep networks via gradient-based localization. Proceedings of the IEEE International Conference on Computer Vision (ICCV).

